# Out-of-pocket expenditure on prenatal and natal care post Janani Suraksha Yojana: a case from Rajasthan, India

**DOI:** 10.1186/s41043-016-0051-3

**Published:** 2016-05-20

**Authors:** Dipti Govil, Neetu Purohit, Shiv Dutt Gupta, Sanjay Kumar Mohanty

**Affiliations:** 1 Department of Population Policies and Programs, International Institute for Population Sciences, Govandi Station Road, Deonar, Mumbai, 400088 Maharashtra India; 2 The IIHMR University, 1, Prabhu Dayal Marg, Near Sanganer Airport, Jaipur, 302029 Rajasthan India; 3 Department of Fertility Studies, International Institute for Population Sciences, Govandi Station Road, Deonar, Mumbai, 400088 Maharashtra India

**Keywords:** OOPE, Antenatal care, Delivery care, Cash assistance scheme, India

## Abstract

**Background:**

Though Janani Suraksha Yojana (JSY) under National Rural Health Mission (NRHM) is successful in increasing antenatal and natal care services, little is known on the cost coverage of out-of-pocket expenditure (OOPE) on maternal care services post-NRHM period.

**Methods:**

Using data from a community-based study of 424 recently delivered women in Rajasthan, this paper examined the variation in OOPE in accessing maternal health services and the extent to which JSY incentives covered the burden of cost incurred. Descriptive statistics and logistic regression analyses are used to understand the differential and determinants of OOPE.

**Results:**

The mean OOPE for antenatal care was US$26 at public health centres and US$64 at private health centres. The OOPE (antenatal and natal) per delivery was US$32 if delivery was conducted at home, US$78 at public facility and US$154 at private facility. The OOPE varied by the type of delivery, delivery with complications and place of ANC. The OOPE in public health centre was US$44 and US$145 for normal and complicated delivery, respectively. The share of JSY was 44 % of the total cost per delivery, 77 % in case of normal delivery and 23 % for complicated delivery. Results from the log linear model suggest that economic status, educational level and pregnancy complications are significant predictors of OOPE.

**Conclusions:**

Our results suggest that JSY has increased the coverage of institutional delivery and reduced financial stress to household and families but not sufficient for complicated delivery. Provisioning of providing sonography/other test and treating complicated cases in public health centres need to be strengthened.

## Background

Since the millennium declaration, the global, national and regional efforts to improve health-related millennium goals (reduction of maternal mortality and child mortality) in developing countries were intensified. Improving the maternal health services become the leading strategy to reduce maternal and child mortality. Several innovative programmes from many developing countries including the conditional cash transfer schemes in India (Janani Surakhya Yojana), Nepal (safe delivery incentive program) and Bangladesh (maternal health voucher schemes in Bangladesh) were introduced to increase the demand for maternal health services specifically among poor and marginalised. A large and growing literature from these countries suggests the functioning, utility and limitations of these programmes [1–18]. Though these programmes were context specific and differ by design and implementation strategy, they had the common goal to reduce the out-of-pocket expenditure on maternal care and increase the access to maternal services among the poor and marginalised. A number of evaluation studies reported the spectacular success of the various schemes in increasing access to maternal services [9, 11, 16, 17, 19]. Despite these programmes in place, the progress in health-related Millennium Development Goals is slow and largely uneven across regions, income groups and by social attributes [20–23].

India accounts one fourth of child mortality and one fifth of maternal mortality worldwide [24, 25]. The inequalities in health outcome and health care utilisation are large across geography, economic status and education [26–29] and largely resulting from financial, cultural and social constraints. In India, in the year 2004–2005, about one fourth of the mothers who did not deliver at health centre reported cost as barrier for not availing the services [30]. Especially poor households consider spending a huge amount on child-bearing deterrent, leading to low health care utilisation among them. This is also corroborated by the fact that the out-of-pocket expenditure on health care accounts about two thirds of the total health expenditure [31] and health expenditure is often catastrophic [32, 33]. Though the maternal health services were free in public health centres, there were charges on medicine, bed, user fees and bribe [34–37].

Recognising that an increase in the level of utilisation of health care for child birth may lead to a reduction in the maternal mortality and neonatal mortality, the Ministry of Health and Family Welfare, Government of India, launched conditional cash transfer scheme, i.e., *Janani Suraksha Yojana (JSY)* (*Janani* denotes to *mother*, *Surakasha* for *protection/safety* and *Yojana* means *scheme*), to address the delays of decision-making, transportation and access to services. The JSY is a flagship intervention programme under National Rural Health Mission (NRHM), launched in April 2005. The JSY scheme covered transport cost, delivery cost and incentive to Accredited Social Health Activists (ASHAs) for motivating women to opt for institutional delivery. It also facilitated public-private partnerships by providing accreditation to private hospitals/nursing homes for delivery services. The cash incentive of INR 1400 (US$30) to recent mothers was given at the time of discharge from the hospital after verifying all the records.

Since the implementation of NRHM, the institutional deliveries in India had increased from 41 % in 2005–2006 to 81 % in 2013–2014 [30, 38] and the infant mortality declined from 58 infant deaths in 2005 [39] to 35 infant deaths per 1000 live births in 2013–2014 [38]. The maternal mortality ratio (MMR) had declined from 254 in 2004–2006 to 167 per 100,000 live births in 2011–2013 [40, 41]. A number of state specific studies were also undertaken to inform the functioning and progress of NRHM in general and JSY in particular [15, 42–45]. The gap (for institutional deliveries) between low and high performing states started reducing despite the fact that there was no differential change in the availability and access of any health facility [43]. This indicated that people were able to avail skilled care at the time of delivery irrespective of the economic status.

Studies on OOPE consistently report higher expenditure for deliveries conducted in private health care centres and for complicated deliveries and caesarean deliveries [32, 46–54]. The OOPE for antenatal care in child-bearing process was also found much higher than delivery care alone [55]. The expenses incurred during child bearing also varied with the place of antenatal care (ANC), indicating that the contact with private facility at any stage of pregnancy will increase cost per delivery [52]. A normal delivery in a health care facility in Nepal was US$64 compared to US$129 per caesarean delivery while excluding opportunity cost [54]. Similarly, in the case of India before NRHM, the cost per delivery in a public and private health institution was US$25 and USD$104, respectively, whereas average cost at antenatal care was US$10 [32]. For Bangladesh, these costs were US$85 and US$181 [56]. The cost per complicated delivery was significantly higher in Tanzania, Africa, Kenya, Burkina Faso and Lao PDR [46, 50–52].

Though a number of studies have examined on the coverage of maternal care services, differentials in OOPE by type of health facility, there are not many studies on cost of antenatal care and the coverage of JSY incentives. The aim of this study is to examine the variation in out-of-pocket expenditure in accessing maternal health services (antenatal and delivery care) and the extent to which the JSY incentives covered the burden of cost incurred.

The paper has been conceptualised with the rationale. First, the cost was one of the major barriers in availing the antenatal and natal care among poor and marginalised. This has particular bearing in the state of Rajasthan that has higher infant and maternal mortality than national average and large variation in maternal care utilisation among different population sub-groups within the state. Second, because of poor quality of the services at public health care institutions, utilisation of maternal health services from private sector increased over a period of time, which had a significant burden on the economic condition of households across various socio-economic groups. Also, women switch the service provider from public to private and vice versa during pregnancy and child birth. No attempt has been made to capture this pattern of service utilisation. Third, it is important to document to what extent the large investment on maternal care under the NRHM helped in the reduction of cost for end users. This is generally of great interest to policy makers and planners for evidence-based policy-making.

## Methods

This paper is based on a cross-sectional study conducted in Rajasthan during April to May 2011. The state of Rajasthan with a population of 69 million in 2011 [57] had the second highest maternal mortality ratio in the country (255 per 100,000 live births) [41] and at low level of socio-economic development. The study was conducted in the four districts of Rajasthan, namely, Udaipur and Banswara (tribal districts—with 49.7 and 76.4 % tribal population, respectively), and Sikar and Sawai Madhopur (nontribal districts). A multi-stage sampling was used to select the sample. From each selected district, two blocks were identified based on the highest and lowest proportion of institutional deliveries. Data on number of deliveries was obtained from Pregnancy and Child Tracking System of Government of Rajasthan (Management Information System—MIS). In this system, information from each village of Rajasthan is being recorded with the help of ground level health care workers. Government claims to maintain the data on all the pregnancies occurred in Rajasthan. From each of the blocks, two primary health centres (PHCs) were selected based on their performance on institutional deliveries (highest and lowest). From each PHC (in total 16), two villages were selected randomly. All the women who gave births (JSY beneficiaries and non-beneficiaries) 1 year prior to survey (during April 2010–March 2011) were interviewed from 32 villages of four districts. A list of women who gave birth during the period was obtained from ASHA (Accredited Social Health Activist) and ANM (Auxiliary Nurse Midwife) of the village. A total of 424 women were successfully interviewed under the study. A structure interview schedule was prepared for data collection and pre-tested in a village before final survey. Data was collected on multiple issues related to accessibility, availability and utilisation of services including cost incurred during pregnancy, delivery and post-delivery and money received under JSY.

The direct cost incurred during childbearing (pregnancy and delivery) was termed as out-of-pocket expenditure (OOPE). The OOPE includes (a) expenditure during antenatal care such as registration fees, doctor’ fee, medicine, tests, sonography and transportation and (b) expenditure incurred during delivery—registration, transportation, doctor, medicine, tests, bed and food. The amount spend on bribe and gift was not included in the study. The cost incurred during postnatal care primarily included the cost on child health, therefore not included in the expenditure on maternal care. The OOPE in the paper is synonymous to the total expenditure incurred during a pregnancy/delivery irrespective of JSY amount. There were a few cases where the expenditure was very large. To reduce the variation, we have levelled the value at 95 % level; e.g., those 5 % women with higher than OOPE (antenatal care) of US$179 were kept at US$179, and similar approach was followed for computing expenditure during natal care. The total OOPE was adjusted to average US$ in the year 2010–2011 (US$1 = 46.2 INR) [58]. The term normal delivery is defined as the delivery without any complication.

Descriptive statistics (mean, confidence interval, per cent distribution) was carried out to understand the differentials in OOPE on antenatal and natal care. A log linear regression model was used to understand the significant predictors of OOPE. Log of OOPE (continues variable) is the dependent variable. The independent variables are both continuous and categorical. The continuous variables are education, age, duration of stay in hospital and birth order. The categorical variables are BPL status of the family, complications during pregnancy and delivery, received JSY incentives and place of ANC.

### Ethics

Ethical clearance for conducting the study was taken from the ethical board of Indian Institute of Health Management Research (IIHMR), Jaipur. Verbal informed consent was taken from women participating in study with the assurance that confidentiality will be maintained and information obtained for this study will not be used for any other purposes except for research.

## Results

Table 1 presents the descriptive statistics of the study population. The mean age of the women was 25.7 years, and about half of them were illiterate. Half of the women belonged to schedule caste/tribe, and more than half were working for wages or kind. About one third of the women were living below poverty line (BPL). Most of the women had received at least three or more antenatal check-ups with a mean of 3.5. The distribution of women by source of antenatal care showed that 43 % women availed the antenatal check-up exclusively from public facilities, 21 % from private facilities and 36 % from both public and private facilities. However, the coverage of full antenatal care (3 check-up + 2 tetanus toxoid (TT) injections + received 90 iron folic acid (IFA) tablets) was only 24 %. On the other hand, the proportion of institutional deliveries was 83: 62 % delivered in public health facilities, 17 % at private health facilities and 3.5 % at accredited private health facilities. Of total, 66 % of the women received incentives under JSY. Almost all women who delivered at public health facilities received JSY incentives (96 % had already received and 4 % were about to receive at the time of survey). More than half of the respondents (57 %) had received any postnatal care (PNC).

**Table 1:** Sample profile of the women covered under the study in Rajasthan, 2011 (*N =* 424)

Background characteristics	Percentage	*n*
Literacy rate	44.8	190
Caste		
Schedule caste	13.0	55
Schedule tribe	37.7	160
Other backward saste	34.7	147
Others	14.6	62
Working	52.8	224
Households possessing BPL card	33.3	141
Mothers received 3 or more ANC	76.9	326
Full antenatal care (3 ANC + 2 TT + received 90 IFA)	24.3	103
Place of antenatal care		
No ANC	1.2	5
Public facility	42.9	182
Private facility	20.5	87
At both public and private facility	35.4	150
Place of delivery		
Public facility	62.3	264
Accredited private facility	3.5	15
Private facility	17.2	73
Home	17.0	72
Mothers received incentives under JSY	65.8	279
Mothers received postnatal care	57.0	241

**Fig. 1:**
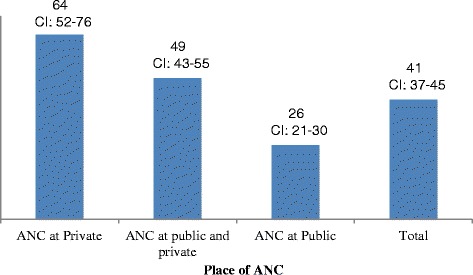
OOPE (in US$) on antenatal care by place of antenatal care, Rajasthan, 2011

Figure 1 presents the out-of-pocket expenditure (OOPE) on antenatal care by place of antenatal care. It may be mentioned that the OOPE on antenatal services includes costs incurred on registration, fee to the doctor, medicines, blood and urine tests, sonography and transportation. The mean OOPE on antenatal care was US$41 (95 % CI 37–45), US$26 in public health facilities and US$64 for those availed services at private health facilities. Women, who availed antenatal care services exclusively from private facility, spend nearly two and a half times higher than women who availed services exclusively from public health facility. Women who availed at both public and private health facilities spent UD$49.

Though all women reported the total cost on antenatal care, only 23 % could provide the expenditure incurred on various services during antenatal care. The share of medicine in OOPE was 59 % followed by sonography (18 %), blood tests (8 %), transporation (8 %) and doctor consultation (7 %). It varied almost in the similar proportion among women who received antenatal care exclusively from public facility, private facility or from both public and private facilities. If women received ANC from public sector, they spent 53 % on medicine, 19 % on sonography, 9 % on tests, 12 % on transportation and 7 % on doctor’s fee of total OOPE. And, this proportion was 60, 17, 7, 9 and 7 %, respectively, if they went to private sector for antenatal care.

### OOPE on delivery care

The OOPE on delivery care and antenatal and delivery care (together) by place delivery is presented in Table 2. The OOPE on delivery was US$44, US$51 for institutional deliveries and US$10 for home deliveries. The OOPE at public health centre was estimated at US$39 compared to US$88 in private health facilities and US$72 for accredited private health facilities. By considering the expenditure on antenatal and delivery care, the total OOPE expenditure was estimated at US$85. The OOPE expenditure on antenatal and delivery care in public health centre was significantly lower (US$78 per delivery) compared to US$154 in private health centre and US$133 at accredited private health centre.

**Table 2:** OOPE^*^ (in US$) on delivery care and antenatal and delivery care by place of delivery in Rajasthan, 2011

Place of Delivery	Expenditure (in US$) during				*N*
	Delivery care		Antenatal and delivery care		
	Mean	95 % CI	Mean	95 % CI	
Institution^a^	51	46–56	96	87–105	352
Public facility	39	34–45	78	69–87	264
Accredited private facility	72	44–101	133	91–176	15
Private facility	88	75–102	154	131–176	73
Home	10	7–12	32	25–40	72
Total	44	39–49	85	78–93	424

^*^JSY incentive has not been deducted from OOPE

^a^Institutional includes private, public and accredited private health facilities

A pregnant woman may avail antenatal care services from private health care facility and deliver at a public health facility or vice versa. This behaviour would affect the OOPE occurred during the child-bearing process. Percent distribution of women by place of antenatal care and delivery is shown in Table 3. Among women who delivered at public health centre, half of them obtained antenatal care services from public facility and nearly one third of them availed antenatal care services both from public and private facility. It is interesting to note that among women who delivered at private facility, 48 % of them received antenatal care services from both public and private health facility. Further analysis of OOPE revealed that the expenses incurred varied significantly with the place of antenatal care and delivery. When all antenatal check-ups were availed from public health facility, and the delivery too was conducted at public health facility, the average OOPE was US$59. However, when the place of delivery was private facility, the average expenditure increased to US$97 and exceeds spending of US$38 over the amount spent on delivery at public health facility (Table 3).

**Table 3:** Place of delivery by place of ANC (%) and OOPE^*^ by place of delivery and antenatal care (in US$) in Rajasthan, 2011

Place of antenatal care	Place of delivery					
	Percentage			OOPE (in US$)		
	Public facility	Private facility	Home	Public facility	Private facility	Home
Public facility	52.7	14.8	44.9	59 (CI 50–69)	97 (CI 39–154)	21 (CI 12–30)
Private facility	13.4	37.5	27.5	107 (CI 75–140)	183 (CI 149–217)	32 (CI 17–48)
Both public and private facility	34.0	47.7	27.5	97 (CI 82–111)	141 (CI 115–167)	55 (CI 39–72)

^*^JSY incentive has not been deducted from OOPE

When a woman availed antenatal services at private health facility but chose to deliver at public facility, the OOPE was US$107. As expected, the respondents who received ANC services at private and delivered at private health facility spend US$183. OOPE among women who availed ANC services both from public and private health facility or delivered at private revealed that the contact with private health facility at any stage of child-bearing process increased the OOPE.

#### Complications and OOPE

Table 4 describes the OOPE on antenatal and delivery care by complications. In general, the OOPE increases with complications both during pregnancy and delivery. For example, a normal delivery without any complication at home costs an average of US$14, US$44 at public facility and US$92 at private facility. Complications during pregnancy alone had contributed to increase in expenditure. Respondents who suffered from complications during pregnancy and delivered at public health facility had spent US$72. Women who suffered from complications both during pregnancy and delivery spent an average of US$145 in public facility delivery and US$197 for private health facility delivery.

**Table 4:** OOPE^*^ (in US$) on antenatal and delivery care by pregnancy and delivery complications^#^ and place of delivery in Rajasthan, 2011

Complications		Place of delivery			
		Institutional	Public	Private	Home
Normal deliveries	Mean	52	44	92	14
	95 % CI	41–62	33–55	61–124	8–20
	*N*	83	70	13	29
Complications during pregnancy but not during delivery	Mean	90	72	141	44
	95 % CI	80−100	63−81	118−164	33−56
	*N*	189	140	49	36
Complications during pregnancy and delivery	Mean	163	145	197	48
	95 % CI	139−187	124−173	152−243	13−84
	*N*	76	50	26	7
Complications during pregnancy or delivery	Mean	110	90	160	45
	95 % CI	99−120	80−101	138−183	34−55
	*N*	269	194	75	43

^*^JSY incentive has not been deducted from OOPE

^#^the number of cases was less (four each in public and private facility delivery) in the case of no complication during pregnancy but complications during delivery, therefore, dropped from analysis

#### Share of JSY incentives on cost of antenatal and delivery care

**Fig. 2:**
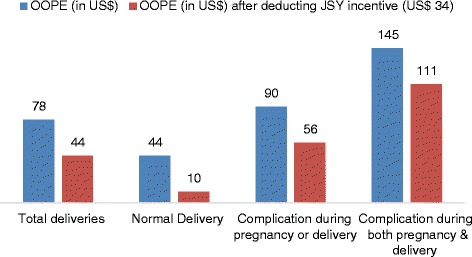
Cost (in US$) per delivery (OOPE and after deducting JSY incentives) in public facilities in Rajasthan, 2011

Figure 2 provides OOPE and JSY incentives provided by government per delivery. As stated elsewhere, the OOPE in public facility was US$78. Further, it was US$44 and 145 for normal and complicated delivery, respectively. Since the government was reimbursing US$34 directly to the beneficiary as JSY incentive, it was deducted from OOPE to get the actual expenditure made by the beneficiary per delivery. After deducting JSY incentives, OOPE per delivery was estimated at US$44. The government share accounts 44 % of OOPE per delivery, 77 % for normal delivery, 38 % for delivery that had complications during pregnancy or delivery and 23 % for those deliveries who had complications both during delivery and pregnancy.

#### Socio-economic differentials in OOPE

Table 5 provides the socio-economic differentials in institutional delivery and OOPE. Comparatively larger proportion of younger women opted for institutional deliveries than older women. After deducting JSY incentives the mean OOPE of younger women (below 25 years) was US$57 compared to US$48 among women aged 25 years and above (US$57 vs. US$48). Similarly, women with general caste spend almost double than other caste women even after deducting JSY incentives. Nearly two-third women from BPL and 59 % from APL delivered at public health centre. Among these, a BPL family spends US$56 on antenatal and delivery care compared to US$91 among APL families. JSY incentives were received by both the sections. JSY incentives reduced the total cost by 47 % among BPL families and 32 % among APL families. Almost the same proportion of literate and illiterate women delivered in public facilities; their expenditure on antenatal and delivery care varied significantly (for illiterate US$68; for literate US$91). Results were similar by husband’s literacy status; in the case of literate husband, the spending on antenatal and natal care was almost double than when husband was illiterate. JSY incentives shared the expenditure to a greater extend in the family where husband was illiterate, even more than where a woman was illiterate. As the birth order increased, the proportion who delivered at institution and the expenditure on antenatal and delivery care reduced. Women, who delivered for the first time, spend US$90 as compared to women who delivered their second, third and more order births (US$82 and 62, respectively). Therefore, the reduction in the OOPE due to JSY was greater in the higher-order birth than in the lower-order birth. Nearly 60 % of the women who delivered their children in the hospital stayed for more than 48 h. Women who stayed for more than 48 h, 82 % of them delivered in public facilities. Increase stay in hospital also increased the expenditure on child birth. Those who stayed in public hospital for 48 h paid US$29 additional as compared to woman who stayed less than 48 h (US$59 vs. 88). This gap widened (US$82), when the delivery was conducted at private facility. The reduction in OOPE due to JSY was less among women staying for longer duration.

**Table 5:** Place of delivery and expenditure by background characteristics. Percentage distribution of women by place of delivery, mean OOPE (in US$) and JSY incentive as percentage of total cost per delivery by background characteristics in Rajasthan, 2011

Background characteristics	*N*	Percentage of women delivered at public facilities	Percentage of women delivered at private facilities	Expenditure during antenatal and natal care by place of delivery				
				Public facility			OOPE in private facility	OOPE in home delivery
				Total cost	Actual OOPE less of JSY incentives	Incentives as % of total cost		
Age								
Less than 25	206	65.0	20.9	84	57	31.7	168	44
25 and above	218	59.6	20.6	72	48	33.6	134	24
Caste								
General	62	54.8	35.5	116	82	28.8	177	32
Others	362	63.5	18.2	72	45	37.9	141	32
Economic status								
BPL cardholder	141	69.5	12.8	56	30	46.4	101	22
APL	283	58.7	24.7	91	61	32.4	163	38
Type of family								
Nuclear	111	55.8	15.3	92	63	31.5	162	24
Joint/extended family	313	64.6	22.7	74	46	38.0	147	39
Literacy								
Illiterate	234	64.9	12.4	68	42	38.6	134	26
Literate	190	59.0	31.1	91	60	33.8	158	35
Work status								
Not working	200	72.0	20.5	73	45	38.3	142	43
Working	224	53.6	21.0	71	55	22.8	157	23
Husband’s literacy								
Illiterate	84	60.7	14.3	48	23	51.3	117	24
Literate	340	62.6	22.3	85	56	34.1	155	36
Birth order								
One	140	66.5	22.9	92	61	33.2	165	50
Two	124	58.2	27.5	82	54	34.0	149	29
Three and above	160	61.9	13.8	62	36	42.2	149	27
Place of ANC^a^								
Public facility	182	75.9	7.1	59	34	42.4	97	21
Private facilities	87	40.3	38.0	107	76	29.7	183	32
Both public and private facilities	150	59.3	28.0	97	65	33.0	141	55
Duration of stay in hospital after delivery^b^								
Less than 48 h	145	65.5	34.5	59	34	43.3	115	NA
More than 48 h	207	81.6	18.4	88	59	33.5	197	NA
N	424	279	145	264	264	264	88	72

^a^Includes home deliveries

^b^Only institutional deliveries

#### Determinant of OOPE

To understand the determinants of OOPE, a log linear regression model is used for all nonzero cases. Further log linear regression model is suitable for OOPE as the OOPE is skewed in nature. All variables except age and place of ANC (private) are significant in the model (Table 6). Educational attainment, duration of stay and complications are positively associated with OOPE. For example, with increase in educational attainment by 1 year, the OOPE is likely to increase by 2 %. The APL households are likely to incur 35 % more OOPE than BPL households. Those women experienced pregnancy complications are likely to spend 61 % more than normal deliveries. These results are in agreement with bivariate analyses and in expected direction. Those who received JSY were likely to spend 46 % less than those not under JSY categories. Similarly, those who had ANC at public hospital were less likely to spend more compared to others. The *R*^2^ value was .408 indicating that 41 % variation in the model is being explained. The F statistics (25.7) was significant, indicating the overall significance of the model.

**Table 6:** Determinant of out-of-pocket expenditure (OOPE): dependent variable—log of out-of-pocket expenditure

Independent variables	Unstandardized beta coefficients	Sig.	95.0 % CI	
			Lower limit	Upper limit
Age (in completed years)	−.010	.477	−.037	.018
Education (in completed years)	.020	.047	.000	.041
Birth order	−.084	.214	−.217	.049
Duration of stay in the hospital (in hr.)	.005	.000	.003	.006
Economic status of family (BPL®)				
APL	.353	.001	.149	.557
Complications during pregnancy/delivery (No®)				
Yes	.612	.000	.392	.832
Received JSY incentives (No®)				
Yes	−.458	.000	−.691	−.225
Place of ANC—public (No®)				
Yes	−.544	.000	−.753	−.334
Place of ANC—private (No®)				
Yes	.095	.468	−.160	.351
(Constant)	7.833	.000	7.127	8.538

## Discussion

In India, enormous efforts were made by the government, nongovernmental organisations and bilateral and multi-lateral donors to increase the level of skilled birth attendance while launching or supporting dedicated policies and programmes since 1990s. In this series, National Rural Health Mission emerged as a milestone. About two third of national health budget is being spent on NRHM, and the NRHM is said to bring considerable changes in whole health care delivery system in India especially in reducing infant and maternal mortality. However, the national average conceals large disparities across the states and socio-economic group in the country. Numerous studies have shown the socio-economic inequality in the utilisation of health care services, and the increased services do not necessarily benefit the poor and marginalised. Prior to the launch of NRHM, empirical evidences and research studies suggest that the cost of the health care services was one of the major barriers for poor households to avail the services [30, 32, 59–62] especially for child bearing. After one decade of the implementation of NRHM, especially *Janani Suraksha Yojana* (JSY), the largest conditional cash transfer programme in the world [9], a phenomenal increase was observed in institutional deliveries across Indian states [38]. This study attempts to understand the OOPE by pattern of antenatal care and delivery care in post-NRHM period in the state of Rajasthan.

The following are the salient findings of this study. First, we found about three-fourth mothers received three and more antenatal care and nine out of ten women delivered in a health centre. This is significantly higher compared to 41 % coverage of three or more antenatal care and 32 % institutional delivery in Rajasthan in 2005–2006 [30]. It may be mentioned that majority of women in our sample were from scheduled caste and scheduled tribe. Hence, this is indicative that the NRHM may have resulted in positive effect on antenatal care and institutional delivery in the state of Rajasthan particularly among the poor and marginalised. The finding is in line with studies on the impact of JSY [44, 63, 64]. Second, we found varying OOPE on antenatal care by the type of provider. The mean OOPE of antenatal care among those availed from public health centre was US$26, US$64 from private health centres and US$49 for those who availed from both private and public health centres. This was higher than the cost of antenatal care in the state during pre-NRHM period [32] possibly due to increasing contact and awareness on institutionalised maternal care and the price effect. Third, half of the OOPE on antenatal care and delivery care was on medicines followed by sonography/test and transportation irrespective of type of service provider. Fourth, the JSY covered 77 % of the cost for normal delivery and 23 % of the cost of complicated delivery with an average of 44 %. This result of our study was higher than the study conducted by Gopalan et.al., where they mentioned that JSY covered 26 % of the maternal healthcare cost in rural areas [65]. The women, who suffered from complications during pregnancy and delivery, spent about three and a half times higher than those who had a normal delivery. The cost per complicated delivery was also significantly higher in African countries [46, 50].

The overall findings of the study suggest that JSY has been successful in reducing the OOPE of the beneficiaries opting for delivery at public health facility. Since the incentive was the highlight of the scheme, the increase in institutional deliveries could be attributed to the satisfaction with the incentives attached to the scheme [66]. Evidences support that incentives provided under JSY were able to meet the cost incurred by the family for the delivery to some extent. Therefore, the incentives worked in favour of institutional delivery addressing the financial barriers and enhancing utilisation of maternal care services in Rajasthan. Also, the NRHM strengthened the public health care system while addressing the other barriers for poor health care utilisation [67]. NRHM focused on the facilitating environment for safe motherhood, i.e., utmost care and attention provided to pregnant women and newborns by strong health care delivery system, i.e., availability of skilled health personnel, adequate health care facilities, equipment, medicines and emergency care along with the community mobilisation [67].

## Conclusions

Based on the extent of antenatal and natal care coverage in our study, it is evident that most of the women were availing the services. Therefore, it can be said that the government’s intention to encourage mothers to deliver at a health facility by providing incentives along with improvement in health care system is reducing disparity and bringing women to the health care centre. Hence, the cash incentive should continue and extend to sonography/test during pregnancy period and complicated deliveries. Since complicated deliveries are largely carried out at private health centres, provisioning of financial incentive to complicated deliveries irrespective of the type of provider should be considered. The incentives have been able to substantially reduce the financial burden faced by the women who delivers at an institution. Last, the population based survey should not only collect the cost of delivery care but also cost of antenatal care.

### Limitation of the study

The study followed a cross-sectional design due to limited resource and time. The study could not segregate the post-delivery care cost from child care cost, therefore addressed the expenditure related to prenatal and natal care.
